# The speed of adoption of new drugs and prescription volume after the amendments in reimbursement coverage: the case of non-vitamin K antagonist oral anticoagulants in South Korea

**DOI:** 10.1186/s12889-020-08929-6

**Published:** 2020-05-27

**Authors:** Kyung-Bok Son

**Affiliations:** College of Pharmacy, Ewha Womans University, 52Ewhayeodae-gil, Seodaemun-gu, Seoul, 03760 South Korea

**Keywords:** Adoption of new drugs, Reimbursement coverage, Pharmaceutical expenditure, Pharmaceutical policy, South Korea

## Abstract

**Background:**

The speed of adoption of new drugs and frequencies of substitutions leads to changes in health care expenditures as well as patient outcomes. In this study, we aim to understand the speed of adoption of new drugs and their prescription volume in health care institutions and evaluate the impact of policy options to manage pharmaceutical expenditure.

**Methods:**

We conducted a retrospective cohort study of health care institutions prescribing NOACs, including Apixaban, Dabigatran, and Rivaroxaban, to address the speed of adoption and their substitution from October 1, 2010, through December 31, 2015, using the National Health Insurance Service-National Sample Cohort. Two threshold time points, including the extension of reimbursement with the need for the letter of opinion and the withdrawal of the letter of opinion, were noted in this study. Then, we applied a survival analysis to elucidate factors that affected the speed of adoption of NOACs, and interrupted time series analysis to estimate the effect of amendments in reimbursement coverage in prescription volume.

**Results:**

Among 934 health care institutions in a study population, 334 institutions (36%) had prescribed NOACs at least one time during the study period, indicating that health care institutions were conservative in adopting new drugs. However, the speed of adoption was related to the characteristics of health care institution. We also found that prescriptions of NOACs before the withdrawal of the need for the letter of opinion were marginal, and the prescription volume of NOACs was significantly increased after the withdrawal of a letter of opinion.

**Conclusions:**

Health care institutions were conservative in adopting new drugs, and the speed of adoption is not closely related to an increased prescription volume in the short run. Thus, policies that are centered on managing pharmaceutical expenditure should be devised with considering the impact of introducing new drugs in the long run. A letter of opinion, which was devised to manage prescriptions of NOACs, was effective in managing pharmaceutical expenditures in health care institutions, particularly for tertiary institutions. Conversely, the withdrawal of the need for the letter of opinion should be implemented with caution.

## Background

Health systems are struggling with rapidly rising health care expenditures [1–6]. In a pharmaceutical sector, high-priced new drugs are continuously granted marketing authorization and have replaced old and inexpensive drugs [7–9]. However, adoption of new drugs among health care institutions is uneven [10–15]. Not surprisingly, the speed of adoption of new drugs and frequencies of substitutions leads to changes in health care expenditures as well as patient outcomes. Thus, understanding the adoption of new drugs is an interesting research to study. Particularly, non-vitamin K antagonist oral anticoagulants (NOACs) are an ideal example for examining the speed of adoption of high-priced new drugs, the substitution of a lower-priced drug with a new drug, and their implication in managing pharmaceutical expenditures in health systems.

Atrial fibrillation is a common abnormal cardiac heart rhythm. Globally, the prevalence of atrial fibrillation is reported with a wide range of 0.5–2%. The presence of atrial fibrillation is related to ischemic stroke [16]. Patients with atrial fibrillation are prescribed oral anticoagulants (OACs) when they have risk factors. Specifically, Warfarin is prescribed to prevent stroke in patients with atrial fibrillation. Warfarin is a traditional OAC that was approved by the U.S. Food and Drug Administration (FDA) in 1954 and is recommended for patients with atrial fibrillation. However, Warfarin should be prescribed with caution. It has numerous interactions with other drugs and foods and requires frequent periodic international normalized ratio (INR) tests and individualized dose adjustment for each patient. Meanwhile, NOACs have been granted marketing authorization. Specifically, Dabigatran, Rivaroxaban, and Apixaban were approved by the FDA in 2010, 2011, and 2012, respectively. NOACs are considered to be safe and effective as well as convenient to use [17–19]. Furthermore, several observational studies, which were conducted after marketing authorization of these drugs, presented similar or better risk-benefit balance of NOACs compared to Warfarin [17–19].

Rivaroxaban was approved by the Ministry of Food and Drug Safety (MFDS) in South Korea in April 2009. The manufacturer, who wanted the drug to be eligible for reimbursement, submitted the dossier to the Health Insurance Review and Assessment Service (HIRA) in 2009 [20, 21]. However, the reimbursement price of NOACs have been compared to an inexpensive comparator, Warfarin, during the reimbursement decision. For instance, the price of Warfarin 4 mg, which is one of the comparators of Rivaroxaban, was only 72 Korean Won (0.06 USD) in 2010. Thus, the high price of Rivaroxaban proposed by the manufacturer was a hurdle for the drug to be reimbursed. In the end, Rivaroxaban 10 mg was reimbursed in October 2010 with 6030 Korean Won (5 USD). However, the reimbursement coverage of the NOAC was limited to patients with knee or hip joint replacement surgery.

**Fig. 1:**
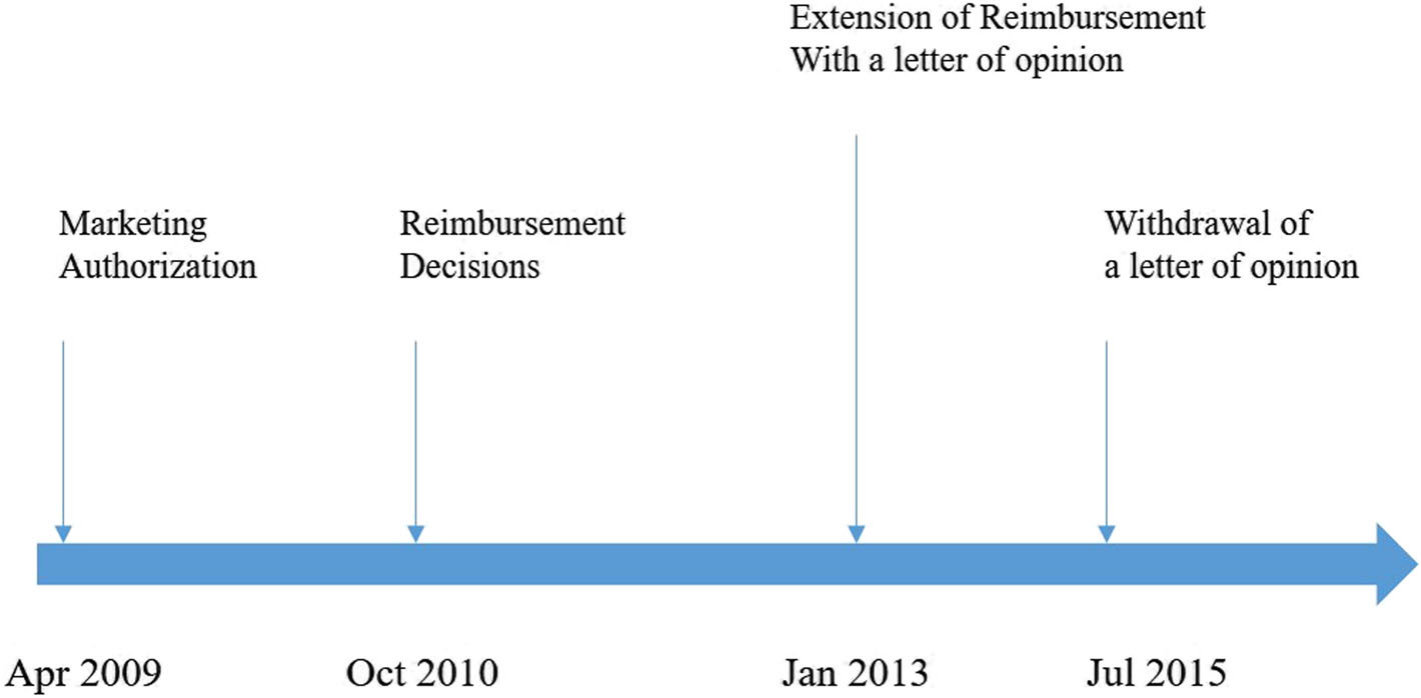
The regulatory approval and reimbursement process of NOACs in the South Korean market

Figure 1 presents the regulatory approval and reimbursement process of NOACs in South Korea. Two threshold time points should be noted after the reimbursement decision: the extension of reimbursement with the need for the letter of opinion and the withdrawal of the need for the letter of opinion. In January 2013, the reimbursement coverage of Rivaroxaban was expanded to patients with nonvalvular atrial fibrillation to reduce their risk of stroke and systematic embolism and to patients with deep vein thrombosis to reduce their risk of recurrent disease and pulmonary embolism. Considering the prevalence of diseases, the expanded reimbursement coverage to patients with nonvalvular atrial fibrillation was the main part of the amendment. It is also realistic to assume that these amendments might cause a sudden increase in pharmaceutical expenditure. The need for the letter of opinion, which is like prior authorization requirements, was devised to manage the prescription volume of NOACs and the pharmaceutical expenditure for NOACs by the government. The HIRA requested that physicians provide a letter of opinion when they prescribe NOACs to patients with atrial fibrillation. Physicians who want to prescribe NOACs for their patients should state in the letter of opinion that the patient cannot be prescribed Warfarin due to hypersensitivity and contraindications and submit the letter to the HIRA. If the letter of opinion is not provided by physicians, then the National Health Insurance Service (NHIS) will not reimburse the prescribed NOACs, implying that patients must pay the total pharmaceutical expenditure. Interestingly, the use of the letter of opinion was withdrawn in July 2015, indicating that physicians could prescribe NOACs to patients with atrial fibrillation regardless of whether Warfarin could be prescribed to the patients. The government assumed that NOACs had presented favorable clinical-effectiveness and thus withdrew the need for the letter of opinion by requesting that manufacturers discount the reimbursement price by approximately 30% [22].

### Reimbursement process for new drugs in South Korea

Reimbursement decisions are necessary if a new drug is to become accessible under the NHIS in South Korea. In this section, we briefly present the reimbursement process for new drugs in South Korea [20, 23].

In 2006, the government adopted a health technology assessment for the reimbursement decision of new drugs. Manufacturers could voluntarily apply for reimbursement after they were granted marketing approval. Note that applying for reimbursement is not mandatory. However, reimbursement decisions are required for a new medicine to penetrate the market. A manufacturer submits the applications and related dossiers to the HIRA, and the HIRA assesses the clinical-effectiveness and cost-effectiveness of the drug. In particular, the Benefit Criteria Advisory Committee reviews clinical-effectiveness, and the Economic Evaluation Subcommittee reviews the economic evaluation. Then, the Pharmaceutical Benefits Committee (PBC) appraises whether the drug can be reimbursed by considering various factors such as the clinical-effectiveness, cost-effectiveness, budget impact, and reimbursement and pricing of the drug in other countries. Note that an explicit threshold for cost-effectiveness is not reported. After the PBC recommends reimbursement, the NHIS negotiates the price and expected volume with manufacturers considering comparable drugs, the number of patients with related diseases, reimbursement criteria, clinical-effectiveness, budget impact, and the reimbursement and pricing of the drug in other countries. Finally, the Ministry of Health and Welfare makes a decision and announces the reimbursement price to the public after the negotiated agreement [23].

Rivaroxaban, which is one of three NOACs, went through the process. In December 2009, the PBC appraised that Rivaroxaban could be reimbursed for patients who have undergone knee or hip joint replacement surgery under the NHIS. Particularly, the appraisal indicated that Rivaroxaban presented clinical-effectiveness for patients with knee or hip joint replacements and acceptable cost-effectiveness when compared with alternatives [24]. Note that the incremental cost-effectiveness ratio was not disclosed in the appraisal document. However, we can conclude that Rivaroxaban presented explicit clinical-effectiveness and tolerable cost-effectiveness for specific patients in South Korea.

In this study, we aim to understand the speed of adoption of new drugs and their prescription volume in health care institutions and evaluate the impact of policy options to manage pharmaceutical expenditure. To this end, we measure the speed of adoption of NOACs, address variations in the speed of adoption, and elucidate factors that affected the speed of adoption at the level of health care institutions. We also analyzed the prescription volume to supplement our understanding in the adoption of new drugs. Specifically, we captured the monthly trends in prescribing NOACs, estimated the effect of policy options to manage pharmaceutical expenditures, including the extension of reimbursement with the need for the letter of opinion and the withdrawal of the need for the letter of opinion, and suggested their implications in managing pharmaceutical expenditures.

## Methods

### Study design

We conducted a retrospective cohort study of health care institutions prescribing NOACs, including Apixaban, Dabigatran, and Rivaroxaban, to address the speed of adoption and their substitution from October 1, 2010, through December 31, 2015. Note that Rivaroxaban was first reimbursed under the national health insurance on October 1, 2010. The reimbursement coverage of NOACs was expanded to atrial fibrillation with the mandatory inclusion of the letter of opinion on January 1, 2013, and the need for letter of opinion for prescribing NOACs was withdrawn on July 1, 2015. We defined the speed of adoption of NOACs as the time difference between the date when NOACs were first reimbursed (October 1, 2010) and the date of the first prescription of NOACs in a health care institution. Additionally, we analyzed the prescription volume to capture the monthly trends in prescribing NOACs and estimate the effect of the withdrawal of the letter of opinion on the prescription volume.

### Study population

We have interests in health care institutions that had prescribed NOACs during the study period. However, some health care institutions that have not yet prescribed NOACs will prescribe NOACs in the future. Thus, we added institutions that had prescribed comparator, Warfarin, in 2010, 2011, and 2012 as eligible subjects to construct a study population.

### Data source

We used the National Health Insurance Service-National Sample Cohort, established by the NHIS [25]. This population-based cohort is composed of 1 million South Korean individuals. In particular, the NHIS constructed a target population of 46,605,433 individuals in 2002 and then randomly selected 1,025,340 individuals from the target population.

The cohort provides four databases on individuals’ insurance eligibility, medical treatments, health care institutions, and general health examinations from January 1, 2002, to December 31, 2015 [25]. Specifically, medical treatment data provided information on the prescriptions, while health care institution data included information on the type of institution and location. We retrieved prescriptions with NOACs from the medical treatment data and merged these data with the health care institution data.

Prescription patterns depend on various factors such as the professional qualification of physicians, ingrained tradition in physicians, market incentive, patient preference, and the availability of treatment guidelines [26–28]. To proximate the professional qualification of physicians, we categorized health care institutions by primary, secondary, tertiary, and quaternary institutions [25]. Primary care institutions include clinic-level medical institutions that provide medical services to outpatients. Secondary and tertiary care institutions include hospital-level medical institutions that provide health services primarily to inpatients. Particularly, secondary institutions were furnished with no less than 30 patient beds, while tertiary institutions had at least 100 patient beds. Quaternary care institutions include superior general hospitals, designated by the Minister of Health and Welfare, that provide medical service requiring a high level of expertise for treating serious diseases. We divided the location into several regions based on the information on administrative district and assumed that location of health care institutions reflected ingrained tradition in physicians which is influenced by patient preference and affordability. For instance, patients who visit health care institutions in rural areas might prefer a lower-priced drug to high-priced new drugs. In the end, four regions were created, including Seoul, metropolitan areas excluding Seoul, urban areas, and rural areas.

### Statistical analysis

This study used descriptive statistics to examine characteristics of health care institutions that had prescribed NOACs and institutions that had not yet prescribed NOACs until December 31, 2015 and employed the χ^2^ test to investigate the associations between prescribing NOACs and types of institution and their location. Furthermore, we divided the institutions that had prescribed NOACs into two groups based on the first prescription date of NOACs: before the withdrawal of a letter of opinion and after the withdrawal of a letter.

In this study, we used two statistical methods to determine the implications of adopting new drugs: a survival analysis and an interrupted time series analysis. First, we applied a survival analysis to measure the speed of adopting NOACs and to elucidate factors that affected the speed of adoption. We calculated the year difference between the date of reimbursement of NOACs (October 1, 2010) and the date of first prescription of NOACs to measure the speed of adoption of new drugs among health care institutions. More specifically, Kaplan-Meier estimates and the Cox proportional hazardous model were applied [29]. Next, we used time series analysis to describe the prescription volume, including NOACs [30]. We presented the monthly trends in prescribing NOACs. Note that the reimbursement coverage of NOACs was extended on January 2013 with the inclusion of the letter of opinion, and the need for the letter of opinion was withdrawn on July 2015. Thus, we estimated the effect of the withdrawal of a letter of opinion on the prescription volume using interrupted time series analysis. Data management and analysis were conducted using R statistical software (version 3.4.3). Statistical significance is noted by p-values less than 0.05.

## Results

### Subjects of the study

Table 1 presents the characteristics of the study population. Among 934 health care institutions, 334 institutions (36%) had prescribed NOACs at least once during the study period. The prevalence of prescribing NOACs is closely related to the types of health care institutions. All quaternary health care institutions, providing a high level of expertise for treating serious diseases, had prescribed NOACs during the study period, while only 80 primary institutions (18%) out of 436 primary institutions had prescribed NOACs. We also confirmed a difference in the distribution of the types of institutions between the prescribed group and the non-prescribed group in the χ^2^ test (p <  0.0001). However, institutions in the prescribed group and the non-prescribed group were evenly distributed in four locations, namely Seoul, metropolitan areas excluding Seoul, urban areas, and rural areas. Specifically, there was no difference in the distribution of the location of the institutions between the two groups in the χ^2^ test (p = 0.2911).

**Table 1:** Baseline characteristics of health care institutions sorted by types and their location

	Before withdrawal *N* = 129	After withdrawal *N* = 205	*p*-value	Prescribed group *N* = 334	Non-Prescribed group *N* = 600	*p*-value
Types						
Quaternary	17	26	0.0612	43	0	0.0001>
Tertiary	54	106		160	71	
Secondary	28	23		51	173	
Primary	30	50		80	356	
Location						
Seoul	26	36	0.6353	62	102	0.2911
Metropolitan excluding Seoul	25	49		74	137	
Urban areas	31	41		72	104	
Rural areas	47	79		126	257	

We also divided the prescribed group into before the withdrawal of a letter of opinion and after the withdrawal of a letter of opinion based on the date of the first prescription of NOACs. Before the withdrawal group was defined as the institutions that had prescribed NOACs before July 1, 2015, while after the withdrawal group was defined as institutions that had prescribed NOACs after July 1, 2015. Among 334 institutions, 129 institutions (38%) were grouped in the before the withdrawal group, while 205 institutions (62%) were grouped in the after the withdrawal group. No significant difference in the types of institutions and their location between two groups was noted.

### The speed of adoption

#### Kaplan-Meier estimates

**Fig. 2:**
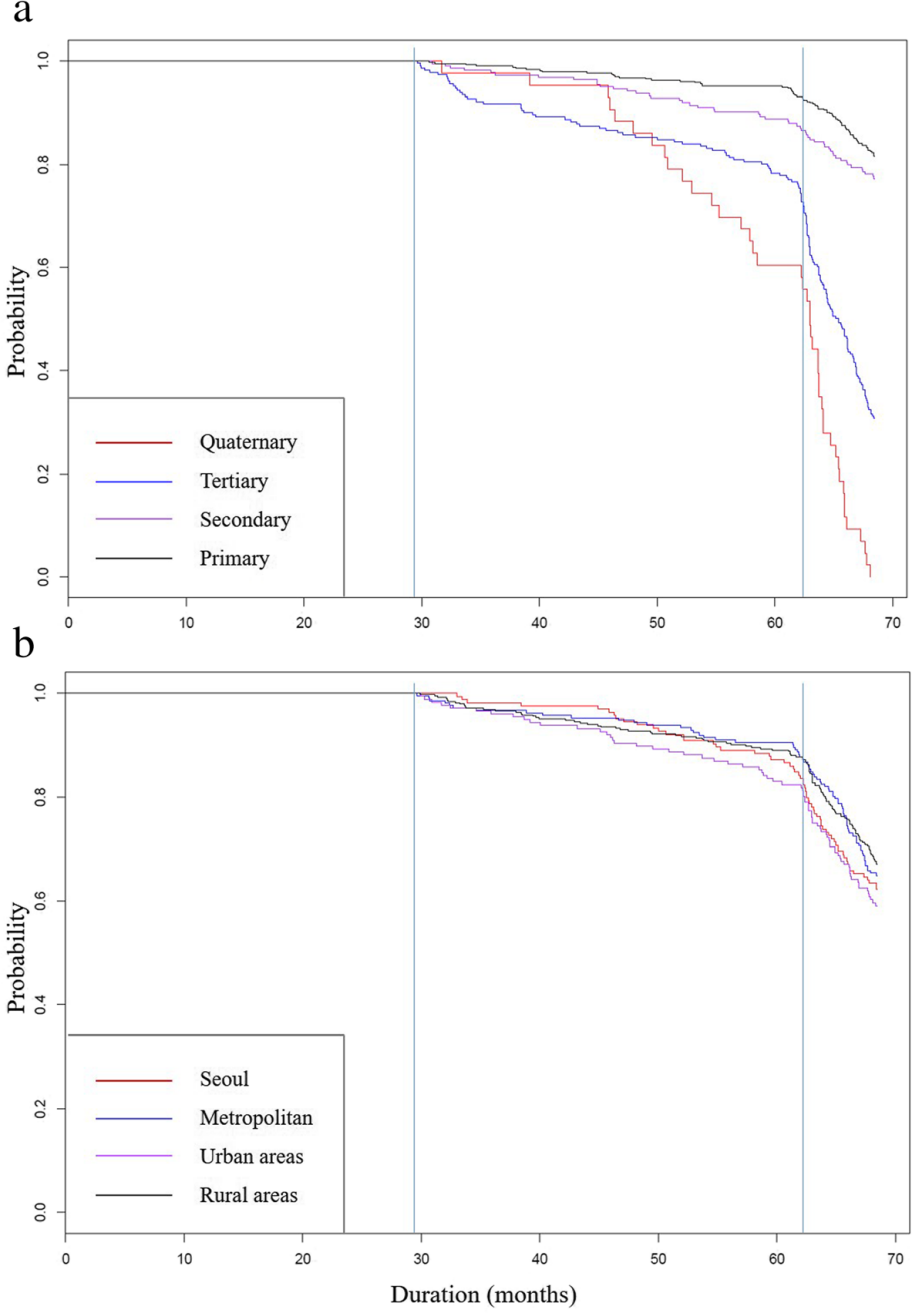
Kaplan-Meier curves for the duration of prescribing NOACs in health care institutions. Note: **a** types of institutions, **b** locations of institutions. Duration starts from the date of the reimbursement decision on October 1, 2010. Durations 29.39 and 61.92 present the date of the extension of reimbursement with a letter of opinion and the withdrawal of the need for a letter of opinion, respectively

Figure 2 provides a descriptive overview of the speed of adoption of NOACs using Kaplan-Meier estimates. The estimates indicate the conditional probability that the institution will prescribe NOACs after a given period [29]. As described in Fig. 1, NOACs were reimbursed on October 1, 2010, (duration 0 month) for limited indications; the indications were expanded to patients with atrial fibrillation and deep vein thrombosis with the need of a letter of opinion on January 1, 2013 (duration 29.39 months); and the requirement for a letter of opinion was withdrawn on July 1, 2015 (duration 61.92 months).

The first graph in Fig. 2 offers a curve with group comparisons in four types of institutions. For instance, after 31.7 months from the date of reimbursement decision, only one quaternary institution prescribed NOACs, while after 62.2 months from the same date, approximately 42% of quaternary institutions had prescribed NOACs. Similarly, after 30.6 months from the date of reimbursement decision, one primary institution prescribed NOACs, while after 61.9 months from the same date, 7% of primary institutions prescribed NOACs. The speed of adoption, which is similar to the slope of a curve, in tertiary health care institutions was the highest in the short term (between duration 30 and duration 45), and the speed of adoption in quaternary institutions was the highest in the long term (between duration 45 and duration 60). We could also find the effect of the withdrawal of the letter of opinion in Fig. 2. In the same figure, the slope of all curve significantly increased on the date after 61.92 months from the first reimbursement date, indicating that many institutions began prescribing NOACs after the withdrawal of a letter of opinion on Jul 1, 2015. The remaining graph in Fig. 2 presents a curve with group comparisons in the location of institutions. In the figure, we could not find deviations in the slope of the curve for each location. However, we could observe the consistent effect of the withdrawal of a letter of opinion in the increased volume of institutions that newly prescribed NOACs across four locations.

#### Cox proportional hazards model

We conducted a multivariate approach to examine the relative impact of the variables on the speed of adoption of new drugs. We fitted the Cox model with two discrete factors: type and location. Table 2 provides interpretations for the variables. Note that a negative coefficient indicates a long time to a prescription (or slow adoption), while a positive coefficient indicates a short time to a prescription (or fast adoption). Thus, the time to prescription for primary, secondary, and tertiary institutions was significantly delayed compared to that of quaternary institutions. However, location was not significantly related to the speed of adoption in our model.

**Table 2:** Results of multivariate Cox model with the duration as outcomes

		Coefficient	Standard Error	*p*-value
Type	(Reference Quaternary)			
	Tertiary	−0.7410	0.1751	< 0.0001
	Secondary	−2.2071	0.2122	< 0.0001
	Primary	−2.4917	0.1947	< 0.0001
Location	(Reference Seoul)			
	Metropolitan excluding Seoul	−0.1986	0.1731	0.251
	Urban areas	−0.1009	0.1747	0.564
	Rural areas	−0.2302	0.1568	0.142

### Prescription volume

#### Trends in prescription

**Fig. 3:**
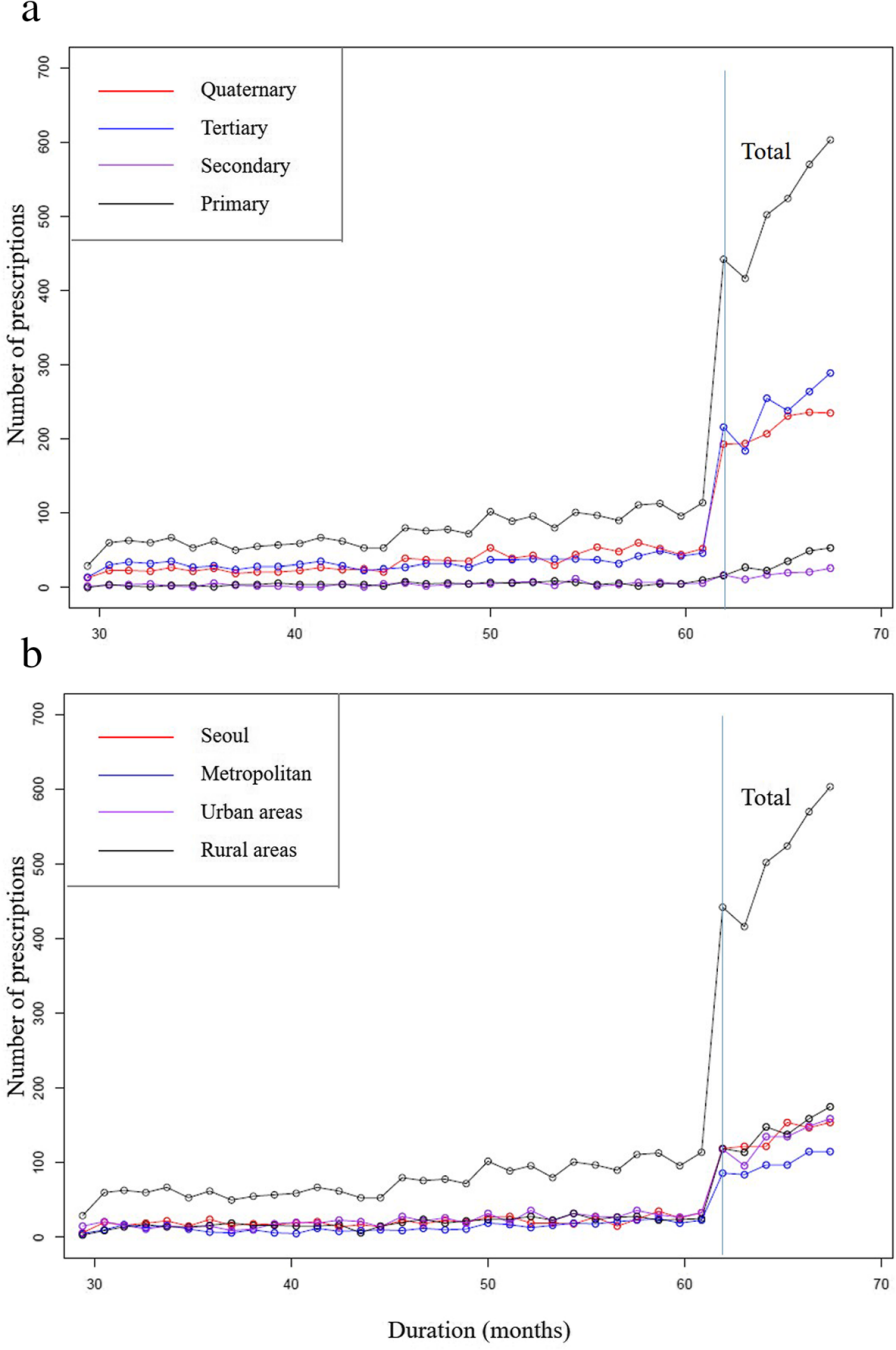
Number of NOACs prescriptions per month from January, 2013 to December, 2015. Note: **a** types of institutions, **b** locations of institutions. Duration 61.92 present the date of the withdrawal of the need for a letter of opinion

Figure 3 provides prescription volume per month from January 2013 to December 2015. The first graph in Fig. 3 presents a curve with group comparison for four types of institutions. We found that prescriptions of NOACs before the withdrawal of a letter of opinion were marginal, specifically less than 100 prescriptions per month. However, the prescription volume of NOACs was significantly increased after the withdrawal of a letter of opinion. In particular, the prescription volume was increased in quaternary and tertiary institutions. The remaining graph presents a curve with group comparisons for locations of institutions. The prescription volume was evenly distributed, and the effect of the withdrawal of a letter of opinion on the prescription volume was consistently observed.

#### Interrupted time series analysis

Table 3 presents the results of the interrupted time series analysis. It is noteworthy that there are three types of coefficients: the time trend before the event, the immediate effect, and the time trend after the event [30]. The coefficient for the time trend before the withdrawal of a letter of opinion means an increase in the monthly prescriptions before July 2015, while the coefficient for the time trend after the withdrawal of a letter of opinion means an increase in monthly prescriptions after July 2015. Finally, the coefficient for the immediate effect means the increase in the prescription volume in right after July 2015. The coefficient before the amendments was marginal (2.17, p <  0.0001). However, the immediate effect of the withdrawal of a letter of opinion was substantial (274.23, p <  0.0001), and the coefficient after the amendments was increased (34.65, p <  0.0001). In particular, the immediate effect was large for tertiary (141.15, p <  0.0001) and quaternary institutions (128.04, p <  0.0001). Similarly, the slope after the amendments was steep for tertiary (16.21, p < 0.0001) and quaternary institutions (9.01, p < 0.0001).

**Table 3:** Results of interrupted time series analysis segmented by types and location

	Time trend before the event			The immediate effect			Time trend after the event		
	Coefficients	Standard Error	*p*-value	Coefficients	Standard Error	*p*-value	Coefficients	Standard Error	*p*-value
All	2.17	0.27	p < 0.0001	274.23	12.89	p < 0.0001	34.65	3.10	p < 0.0001
Quaternary	1.27	0.13	p < 0.0001	128.04	6.50	p < 0.0001	9.01	1.56	p < 0.0001
Tertiary	0.58	0.19	0.0054	141.15	9.21	p < 0.0001	16.21	2.21	p < 0.0001
Secondary	0.14	0.04	0.0066	4.56	2.35	0.0609	2.08	0.56	0.0009
Primary	0.17	0.05	0.0022	0.46	2.42	0.8497	7.34	0.58	p < 0.0001
All	2.17	0.27	p < 0.0001	274.23	12.89	p < 0.0001	34.65	3.10	p < 0.0001
Seoul	0.40	0.11	0.0015	81.84	5.49	p < 0.0001	7.65	1.32	p < 0.0001
Metropolitan without Seoul	0.46	0.08	p < 0.0001	55.14	4.22	p < 0.0001	6.33	1.01	p < 0.0001
Urban areas	0.69	0.13	p < 0.0001	63.57	10.19	p < 0.0001	9.70	1.50	p < 0.0001
Rural areas	0.61	0.10	p < 0.0001	73.66	5.08	p < 0.0001	10.95	1.22	p < 0.0001

## Discussion

Prescriptions of NOACs for patients with acute ischemic stroke and atrial fibrillation have been well documented in literature on clinical pharmacy and medicine [17–19]. Similarly, managing the adoption of NOACs, a first-in-class oral anticoagulant, in various clinical settings is an interesting research area in public health and health financing. However, little is known about how health care institutions approach prescribing new drugs, and how many high-priced new drugs are substituted for old and low-cost drugs in South Korea. We aimed to address the speed of adoption and prescription volume of high-priced new drugs. NOACs, presenting a better risk-benefit balance compared to a traditional and low-priced comparator, Warfarin, are an ideal example to study. We applied a survival analysis to elucidate factors that affected the speed of adoption of NOACs, and interrupted time series analysis to estimate the effect of policy options to manage pharmaceutical expenditures. Based on the empirical analysis, we suggest policy implications for the adoption of new drugs and the management of pharmaceutical expenditures.

### Summary of findings

There are interesting findings in the study. First, we found that health care institutions are conservative in adopting new drugs. Among 934 health care institutions in the study population, 334 institutions (36%) had prescribed NOACs at least once during the study period. However, the speed of adoption was related to the characteristics of the health care institution. For instance, the time to prescription for primary, secondary, and tertiary institutions was significantly delayed compared to that of quaternary institutions. However, location was not significantly related to the speed of adoption. Second, we noted that prescriptions of NOACs before the withdrawal of the need for a letter of opinion were marginal, and the prescription volume of NOACs was significantly increased after the withdrawal of the letter of opinion. Prescriptions of NOACs before the withdrawal of the letter were less than 100 prescriptions per month and increased to more than 400 prescriptions per month after the withdrawal of the letter. Third, we found different trends in the speed of adoption and the prescription volume between quaternary and tertiary institutions. During the study period, most prescriptions were prescribed from quaternary (43%) and tertiary institutions (45%). However, the speed of adoption was faster for tertiary institutions in the short run, while the speed of adoption was faster for quaternary institutions in the long run.

### Being conservative in prescribing NOACs

In our analysis, none of the health care institutions prescribed NOACs immediately after they were reimbursed on October 1, 2010. The first prescription for NOACs was observed after the extension of reimbursement coverage with the need for the letter of opinion (January 1, 2013) in our dataset. Furthermore, we found that most health care institutions (64%) have not prescribed NOACs even after the coverage extended with the need of a letter of opinion, indicating that health care institutions were conservative in adopting new drugs. Being conservative in adopting NOACs could be explained by a variety of factors, such as an intentionally cautious approach to adopting new drugs to ensure patient safety or a lack of awareness of a drug’s introduction [11]. More specifically, physicians have incomplete information on the safety and effectiveness of new drugs [23, 24], and initial clinical trials are often too small to detect rare adverse reactions or demonstrate comparative effectiveness [31]. Furthermore, the HIRA requested physicians to provide a letter of opinion when prescribing NOACs to manage the prescription volume of NOACs in South Korea.

We also confirmed that the speed of adoption varied among the types of health care institutions. In particular, the adoption of NOACs among institutions was in the order of quaternary, tertiary, secondary, and primary institutions: quaternary and tertiary institutions were often faster to adopt new drugs; and primary and secondary institutions were often slower to adopt new drugs. This observation means that high-risk patients might visit quaternary and tertiary institutions or cardiologists at these institutions. Thus, quaternary, and tertiary institutions will be more likely to adopt new medicines earlier compared to primary institutions or primary care physicians.

Our observations were consistent with the previous literature presenting that physicians were conservative in prescribing new drugs and that physician specialty was the most consistent predictors of adopting new drugs [10, 11, 13, 23, 32, 33]. For instance, Anderson et al. (2018) examined the adoption and prescription of three cardiovascular drugs of differing novelty, including Dabigatran, Aliskiren, and Pitavastatin in Pennsylvania in the United States. Note that Dabigatran, which is one of the drugs belonging to NOACs, is a first-in-class drug approved by the FDA in 2010. In the literature, most physicians (73%) did not adopt Dabigatran in the first 15 months after marketplace introduction, and cardiologists were more likely to adopt Dabigatran compared to primary care physicians.

However, O’Connor et al. (2018) reported rapid adoption of novel cancer therapeutics, specifically immune checkpoint inhibitors of programmed cell death 1 protein (anti-PD-1 agents), immediately after FDA approval in the United States [12]. The authors explained that disease severity, preference for novelty, and clinical gains over existing treatments contributed to the rapid adoption of new drugs. Specifically, anti-PD-1 agents presented large gains in efficacy and effectiveness, including better survival in trials and durable responses to treatment, for selected patients [34]. It is also noteworthy that anti-PD-1 agents are widely used to treat many types of solid tumors, including malignant melanoma and non-small cell lung cancer [35].

Furthermore, we presented the difference in the speed of adoption among quaternary and tertiary institutions in this study. In the short term, the speed of adoption among quaternary institutions was like those of primary and secondary institutions, indicating that many quaternary institutions are reluctant to prescribe new medicines immediately after new drugs become available. Thus, the adoption curve for quaternary institutions in Fig. 2 was flat for the first 12 months after the first prescription occurred and significantly dropped after the first 12 months. However, the adoption curve for tertiary institutions smoothly dropped during the same period. Similarly, we analyzed the effect of the location of institutions in adopting new drugs. We found that location of institutions was not related to the adoption of new drugs, indicating the consistent results reported in the previous literature [13, 23].

### Managing pharmaceutical expenditure

We analyzed the prescription volume of NOACs to understand the adoption of new drugs and to manage pharmaceutical expenditure. During the study period, most prescriptions (88%) were prescribed from quaternary (43%) and tertiary (45%) institutions. Interestingly, we found different trends in prescribing NOACs among quaternary and tertiary institutions in our interrupted time series analysis. Before the withdrawal of a letter of opinion, the coefficient of the time trends for quaternary institutions (1.27, p-value < 0.0001) was higher than that of tertiary institutions (0.58, p-value 0.0054). However, the value of the coefficient of the time trends was reversed after the withdrawal of a letter of opinion; 9.01 (p-value < 0.0001) and 16.21 (p-value < 0.0001), respectively. Furthermore, the value of the immediate effect for tertiary institutions (141.15, p-value < 0.0001) was higher than that for quaternary institutions (128.04, p-value < 0.0001). Thus, we can conclude that the withdrawal of the letter of opinion had a stronger effect on tertiary institutions than on quaternary institutions.

This study also captured the effect of introducing a letter of opinion against extension of reimbursement of NOACs. As already demonstrated, a letter of opinion was quite effective in slow adoption of NOACs and managing prescription volume in South Korea. Particularly, all the slopes of the curve after the withdrawal of the letter of opinion increased in Fig. 2, and the prescription volume quite increased after the withdrawal of the letter in Fig. 3. These observations indicate that the introducing the letter of opinion were quite effective in the adoption of NOACs as well as managing prescription volume.

### Study limitations

Our study has some limitations. First, we used a sample cohort and assumed that the cohort was reproducible. However, prescriptions for rare diseases were hard to be captured in the cohort, which was composed of 1 million individuals (2%) selected from South Koreans. Thus, prescriptions that had occurred before the extension of the reimbursement coverage on January 1, 2013 might have been omitted from our dataset. Second, we analyzed the adoption of new drugs in health care institutions. It means that we did not consider the characteristics of patients and their clinical information. Finally, we analyzed NOACs to understand the adoption of new drugs in South Korea, meaning that our findings might not be generalizable to other new drugs. For instance, the adoption of new drugs might vary by the characteristics of the drugs, including disease severity and clinical gains over existing treatments.

## Conclusion

Given the results in the adoption of new drugs and the prescription volume, we could draw some implications in managing pharmaceutical expenditure for new drugs. Health care institutions were conservative in adopting new drugs, and the majority of institutions did not adopt new drugs immediately after their marketplace introduction. Furthermore, the speed of adoption is not closely related to an increased prescription volume in the short run. Thus, policies that are centered on managing pharmaceutical expenditure should be devised with considering the impact of introducing new drugs in the long run. A letter of opinion, which was devised to manage prescriptions of NOACs, was effective in managing pharmaceutical expenditures in health care institutions, particularly for tertiary institutions. Conversely, the withdrawal of a letter of opinion should be implemented with caution from the perspective of health financing.

## Data Availability

The data that support the findings of this study are available from by the National Health Insurance Service but restrictions apply to the availability of these data, which were used under license for the current study, and so are not publicly available. Data are however available from the authors upon reasonable request and with permission of the National Health Insurance Service.

## Abbreviations

FDA: U.S. Food and Drug Administration
HIRA: Health Insurance Review and Assessment Service
INR: International Normalized Ratio
KRW: Korean Won
MFDS: Ministry of Food and Drug Safety
NHIS: National Health Insurance Service
NOAC: Non-Vitamin K Antagonist Oral Anticoagulants
OAC: Oral Anticoagulants
